# Al_2_O_3_/WS_2_ Surface Layers Produced on the Basis of Aluminum Alloys for Applications in Oil-Free Kinematic Systems

**DOI:** 10.3390/ma14247738

**Published:** 2021-12-15

**Authors:** Joanna Korzekwa, Marek Bara, Sławomir Kaptacz

**Affiliations:** Faculty of Science and Technology, Institute of Materials Engineering, University of Silesia in Katowice, 75 Pułku Piechoty 1a, 41-500 Chorzów, Poland; marek.bara@us.edu.pl (M.B.); slawomir.kaptacz@us.edu.pl (S.K.)

**Keywords:** anodization, anodic oxide, anodized materials application, pneumatic actuators nanostructured oxide

## Abstract

The article presents the results of an aluminum oxide layer doped with monolayer 2H tungsten disulphide (Al_2_O_3_/WS_2_) for applications in oil-free kinematic systems. The results concern the test carried out on the pneumatic actuator operational test stand, which is the actual pneumatic system with electromagnetic control. The cylinders of actuators are made of Ø 40 mm aluminum tube of EN-AW-6063 aluminum alloy which is used in the manufacture of commercial air cylinder actuators. The inner surfaces of the cylinder surfaces were covered with an Al_2_O_3_/WS_2_ oxide layer obtained by anodic oxidation in a three-component electrolyte and in the same electrolyte with the addition of tungsten disulfide 2H-WS_2_. The layers of Al_2_O_3_ and Al_2_O_3_/WS_2_ obtained on the inner surface of the pneumatic actuators were combined with a piston ring made of polytetrafluoroethylene with carbon (T5W) material and piston seals made of polyurethane (PU). The cooperation occurred in the conditions of technically dry friction. After the test was carried out, the scanning electron microscopy with energy dispersive spectroscopy (SEM/EDS) analysis of the surface of the cylinder bearing surfaces and piston seals of the pneumatic cylinders was performed. The analysis revealed the formation of a sliding film on the cylinder surface modified with tungsten disulfide, as well as on the surface of wiper seals. Based on the SEM/EDSM tests, it was also found that the modification of the Al_2_O_3_ layer with tungsten disulfide contributed to the formation of a sliding film with the presence of WS_2_ lubricant, which translated into smooth cylinder operation during 180 h of actuator operation. The cylinder with the unmodified layer showed irregular operation after approximately 70 h thereof.

## 1. Introduction

Friction is a complex and nonlinear common phenomenon, dependent on many physical parameters and operating conditions in all mechanical systems. The property of friction is the force of friction which opposes the relative movement of the contacting bodies. The force is tangent to the contact surfaces of the bodies, the direction thereof is consistent with the slip velocity and reveals the opposite sense with respect to this velocity. In some cases, friction is the basis of the operation of mechanisms, in others it is an undesirable phenomenon in which the aim is to minimize its effects. There are many static and dynamic models that describe the friction path [1,2,3,4,5,6,7,8,9,10]. It is necessary to minimize the resistance to movement of, inter alia, pneumatic actuators used in many industrial applications to generate force in reciprocating motion. Cylinder materials must be rust-resistant and capable of handling high temperatures and pressures. The most commonly used materials for the pneumatic cylinders of actuators are: stainless steel, nickel-plated brass, aluminum and steel. The inner side and sometimes the outer side of the cylinder are plated or anodized to reduce wear and corrosion [11]. The choice of material for an air cylinder will depend on the application the cylinder is used for, as well as other factors such as load, stroke length, temperature and humidity of the operating environment [12]. Compared to other reciprocating force devices, such as hydraulic actuators, pneumatic actuators have advantages such as: the possibility of obtaining very high speeds of movement, reliability, low cost, easy installation, easy maintenance and the availability of compressed air in almost all industrial installations. [13,14]. Experimental studies [15,16,17,18] have shown the importance of measuring the friction force of pneumatic and hydraulic actuators, taking into account physical factors such as speed, pressure and other tribological factors. The authors [19] used a thin-film pressure sensor applied to the sliding surface of the piston ring to measure the instantaneous pressure distribution during the sliding of the piston ring on the cylinder liner. The paper [20] presents the importance of proper lubrication conditions with plastic grease and the properties of the seal material for wear resistance. Mazza et al. [21] determined how the geometrical and material properties of the piston rod and piston seals relate to the total friction force between the elements of pneumatic cylinders. The articles [22,23,24,25,26,27,28,29] describe the influence of nanolubricates suspended in lubricating oils on tribological properties. The articles showed that nanolubricates added to oils reduce the coefficients of friction and protect the surfaces of rubbing bodies against wear. The test results describing the tribological cooperation in the technically dry sliding contact have been described, inter alia, by the authors [30], who found that the graphene coating formed on the steel surface reduces both friction and wear compared to the steel surface without the coating. The fretting wear analysis of cylindrical and flat sliding pairs coated with commercial MoS_2_ has been described by the authors [31].

## 2. Materials and Methods

### 2.1. Test Material—Piston Seal

**Figure 1:**
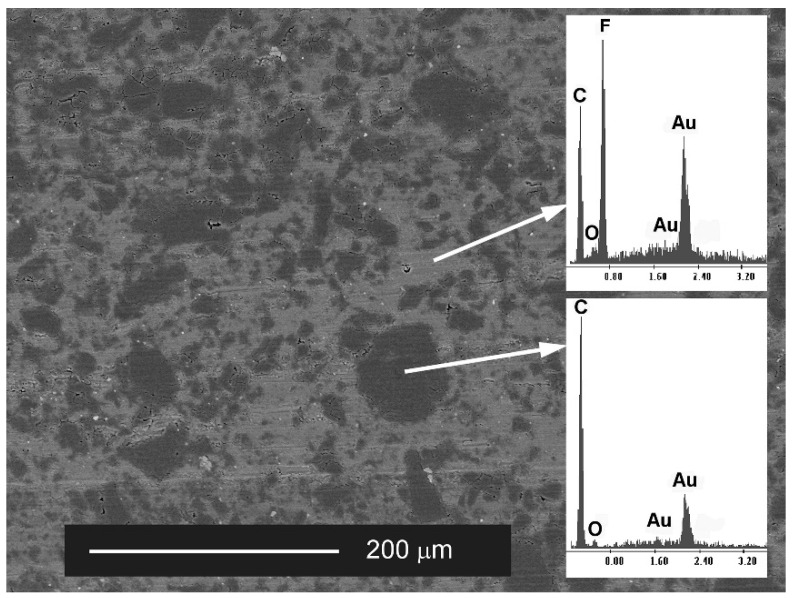
The SEM image of the surface of the T5W plastic with EDS analysis of the chemical composition [35].

The air cylinder actuator was prepared in accordance with ISO 6431. As wiper seals (blue), piston seals were used (CPP “Prema” S.A., Kielce, Poland) [32]. These are polyurethane seals with the highest abrasion resistance which ensures long-term operation in oil-free conditions. A T5W ring is used as a guide seal (black). T5W material is a composite based on PTFE with a dispersion phase in the form of prepared carbon powder (Figure 1 [33]. Addition of carbon to PTFE increases the mechanical resistance, decreases the linear thermal expansion and lowers the abrasive wear. T5W material, due to its low value of friction coefficient is most often used in pneumatic systems of piston-cylinder.

### 2.2. Research Material—The Cylinders of Actuators

**Figure 2:**
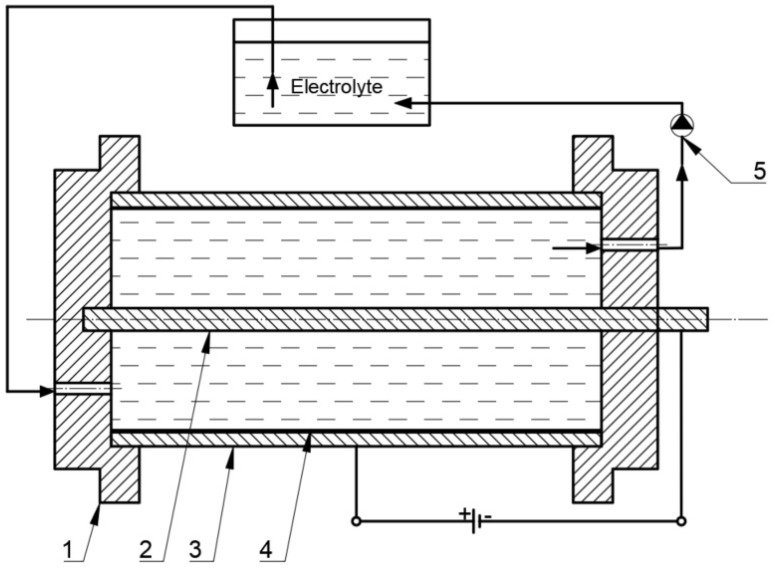
Diagram of the stand for the oxidation of cylinders with forced electrolyte flow circulation: 1—mounting bracket, 2—cathode, 3—cylinder (anode), 4—oxide layer formed, 5—pump.

The material intended for the production of the cylinders of actuators must have good machining properties and be anodized in order to harden the surface and increase corrosion resistance. The basic material for the preparation of the surface oxide layers were cylinders with an internal diameter of Ø 40 × 10^−3^ m and a length of 87 × 10^−3^ m made of EN-AW-6063 aluminum alloy pipe. The inner surfaces of the cylinder surfaces were etched in KOH aqueous solutions for 40 min and HNO_3_ for 10 min, and then hard anodized in a multicomponent electrolyte consisting of an aqueous acid solution: sulfuric, phthalic and oxalic acid (SFS) in accordance with [34]. The hard anodizing process was performed at 3 A/dm^2^ current density and a constant electrolyte temperature of 303 K, on a special laboratory stand for the oxidation of pneumatic cylinders (Figure 2) [35]. In order to ensure uniform layer growth during the oxidation, the electrolyte flow direction was changed. Two cylinders were anodized using a stabilized GPR-25H30D power supply. One layer on the cylinder was obtained by anodizing its inner surface in an SFS electrolyte, the second layer in an SFS electrolyte with the addition of WS_2_ powder (Graphene Supermarket, Megantech grain size <0.4–1 μm), min the amount of 30 g/L. After the anodizing process, the cylinders were rinsed in distilled water for an hour to remove residual compounds from the electrolyte, in which the electrochemical process was carried out.

For the sake of clarity, the article uses the following nomenclature: C1– cylinder with the Al_2_O_3_ layer and C2—cylinder with the Al_2_O_3_/WS_2_ layer.

### 2.3. Research Methodology

**Figure 3:**
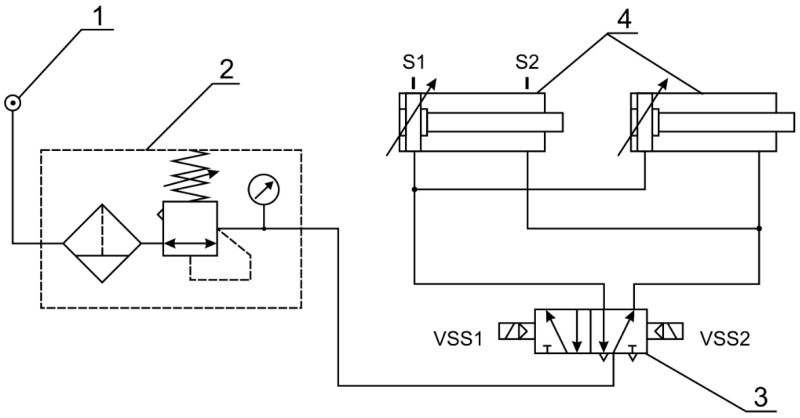
Stand for measuring the operation of actuators: 1—compressor, 2—compressed air preparation block, 3—diverting valve (VSS1, VSS2—valve coils), 4—double-acting actuators (S1, S2—reed sensors).

The test stand consisted of two actuators, the cylinders of which were the test material described above, prepared by electro-oxidation. The laboratory stand for operational testing of pneumatic actuators (Figure 3) is an actual electromagnetically controlled pneumatic system. Additionally, the system has been designed as a stand for testing cylinder bearing surface wear and for testing piston seals in pneumatic cylinders operating in dry friction conditions. The stand consists of pneumatic components: a compressor (Gentilin SRL, Trissino, Italy), a filtering-reducer unit (Prema, Kielce, Poland), two double-acting piston actuators and a 5/2 diverter valve controlled by coils by means of reed sensors installed on the actuator. The test stand enables continuous operation of the actuators in a parallel system.

A Gentilin Compact-air 280 lubricant-free compressor (Gentilin SRL, Trissino, Italy) was used to generate the compressed air. During the tests, the actuators were fed with compressed air at a pressure of 0.5 MPa. The pressure measurements in the actuator chambers were made using Aplisens AS pressure transducers (Aplisens, Warsaw, Poland). In order to measure the temperature in the area of the friction zone, blind holes were made in the actuator cylinders, drilled to a depth equal to the thickness of the cylinder wall. The holes were halfway along the length of the cylinders. K-type thermocouples (Chaki Thermo—Product, Raszyn-Rybie, Poland) were used to measure the temperature. They were placed permanently in the prepared holes. One of the thermocouples for measuring the ambient temperature was placed near the actuators. Both the pressure in the actuator chambers and the temperature were measured using an analogue-to-digital Spider8 transducer. During the measurement of the pressure in the actuator chambers, sampling at 200 Hz was used, and when measuring the temperature, sampling at 1 Hz was used. The acquisition of measurement data was carried out using the Catman 4.5 program. The operational tests were performed during 180 h. The initial speed of both actuators was approximately 0.4 km/h. All seals slidingly cooperating with oxide layers, both before and after the test, were weighed on a WPA 60 scale. After completion of the bench tests, the actuators were disassembled, the cylinders were cut and subjected to further tests. The study of the structure and morphology of the surface of the layers was carried out with the Hitachi S-4700 scanning electron microscope with the EDS Noran Vantage system at a magnification of 35-25000x. For proper observation, the oxide layers were sputtered with carbon using a turbomolecular carbon sputtering machine. The carbon layer enables the bouncing electrons to be discharged and carried away during the research. Surface geometrical structure (SGP) tests were carried out in order to determine the roughness parameters and the geometrical structure of the surface before and after the operational test. Measurements were made by systematic scanning using a Form TalySurf Series 2 50i contact profilographometer. Basic stereometric parameters from the amplitude group and the Abbott–Firestone curve were determined.

## 3. Results and Discussion

### 3.1. Friction in the Layer-Seal System and Pressure in the Cylinder Chambers

**Figure 4:**
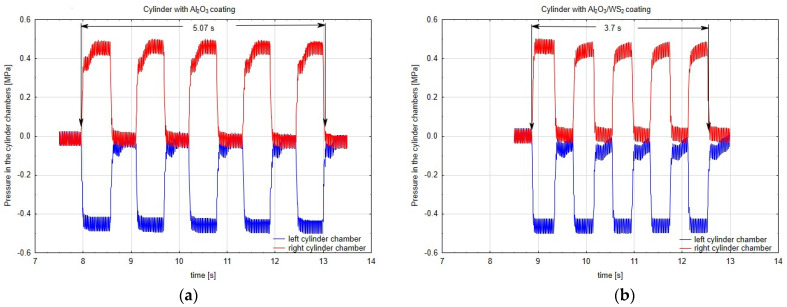
Pressure chart in the cylinder chamber (**a**) with Al_2_O_3_ coating—C1 cylinder, (**b**) with Al_2_O_3_/WS_2_ coating—C2 cylinder.

In the first hours of testing, both actuators powered by air with the same pressure operated evenly. After approximately 80 h of the test, a reduction in the speed of the actuator piston rod with the cylinder surface without modification of WS_2_ was noticed. The speed of the piston rod of this actuator decreased with the test time, which was due to the increasing friction in this actuator. Figure 4a,b respectively present the diagrams of the pressure values in the cylinder chambers of the actuators during the operation of the piston, determined at the end of the test. As an example, a diagram of the pressure distribution during five working cycles of pistons in C1 (Figure 4a) and C2 (Figure 4b) cylindrical liners is shown. As soon as the spool valve is shifted, the pressure in the actuator’s chamber on the left side of the piston (piston extension cycle) rapidly increases to a value of about 0.4 MPa (red diagram). This sudden increase is followed by a flattening of the pressure curve resulting from the adjustable air cushioning system used in the actuators. The applied system causes the braking of the piston in the final stages of the movement, which is manifested in sudden pressure surges visible in the charts, up to the value of 0.5 MPa. When the actuator piston reaches its extreme position, the BSPT system (contactless signalling of the piston position) installed in the actuator piston transmits a signal to the spool valve coil via a contractor. The spool valve is shifted. The pilot pressure is directed to the actuator’s chamber on the right side of the piston (piston retraction cycle). During this time, air from the actuator’s chamber on the left side of the piston is purged to the atmosphere, which is shown in the red diagram as a sharp drop in pressure, down to 0 MPa. In the final phase of this movement, the piston decelerates again, which is manifested by pressure surges. The graphs in blue present the pressure values in the chamber on the opposite side of the piston. As the pressure increases in the chamber on the left side of the piston (red diagram), the pressure in the chamber on the right side of the piston decreases (the transducer, when zeroed before the movement begins, indicates a vacuum). Both graphs show a similar course, which indicates the same nature of the piston’s operation. On the basis of the plotted graphs, the times for five piston work cycles were determined. The time of five cycles for the C1 cylinder was about 5.07 s, while for the C2 cylinder this time was 3.7 s. The movement resistance in the C1 cylinder resulting from the lack of WS_2_ addition to the layer structure resulted in an increase in the working time of the piston by approx. 27%.

### 3.2. Temperature of the Layer-Sealing System

**Figure 5:**
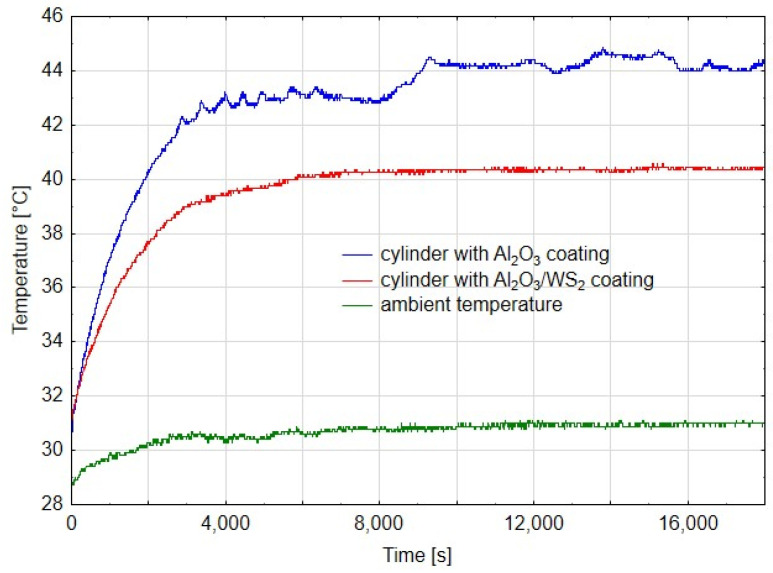
Temperature graph from time after 14 h of cylinder operation.

**Figure 6:**
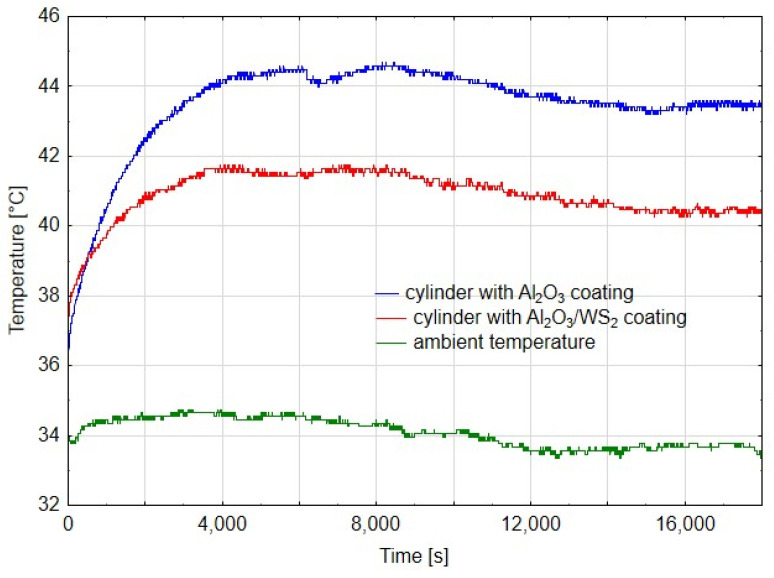
Temperature graph from time after 80 h of cylinder operation.

**Figure 7:**
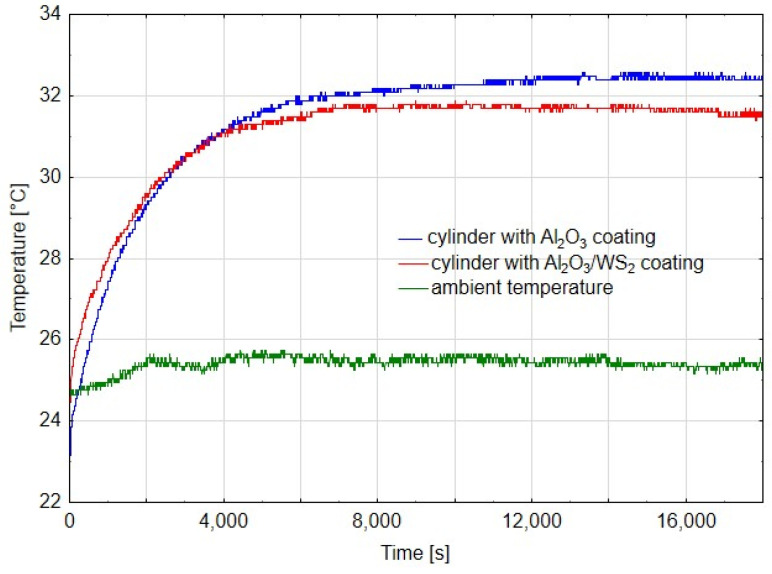
Temperature graph from time after 180 h of cylinder operation.

Figure 5, Figure 6 and Figure 7 show diagrams of temperature measured on cylinders in the area of the friction zone after 14 h, 80 h and 180 h of actuator operation, respectively. The diagrams were made for the first phase of the test, when the actuators worked evenly, after 80 h, when a change in the speed of the C1 actuator piston rod was noticed, and after 180 h in the final stage of the experiment. In addition to the temperature measured on the cylinders, the charts also show the ambient temperature. In diagram 5, the temperature measured for the cylinder C1 is characterized by an irregular course, it is related to the jumps in temperature values in the initial phase of the running-in of two elements. Assuming that the average temperature for C1 was 44 °C, and for C2 40 °C, it can be shown that the temperature in the cylinder C2 was about 9% lower than that in C1. In the case of cylinder temperature measurements after 80 h of operation, where the first differences in the speed of the piston rod movement of both cylinders were noticeable, the average value of the stabilized temperature graph for C1 was 44 °C, and for C2 it was 41 °C, which was a value of approx. 7% lower compared to C1. Both diagrams show a linear course, which suggests the running-in of the sliding friction elements. After 180 h of operation, the difference in cylinder operation temperature was approx. 2%. However, the very irregular cycle of operation of the C1 cylinder, resulting from high resistance to movement of the cylinder piston, excluded it from further tests, which were also completed for the C2 cylinder. As can be deduced from Figure 5, Figure 6 and Figure 7, the ambient temperature also influenced the temperature measurement in the cylinders. For the higher ambient temperature, the temperature measured in the cylinders was also higher. The trend, however, was maintained and therefore, conclusions can be drawn when comparing the temperatures in the area of the friction zone, which was one of the main objectives of the study. The temperature difference between the cylinder C1 and C2 in the first stage of the tests (Figure 5) results from the nature of the cooperation of the cylinder piston seals with the Al_2_O_3_ layer, which the greater resistance to movement than in the case of cylinder C1, generating a higher temperature. The reduction of the temperature difference between the cylinders C1 and C2, in the further part of the tests (Figure 6 and Figure 7), may have two reasons: a change in the character of the running-in tear to the stabilized stage and a change in the speed of the cylinder piston rod C1. The reduction of this speed generated a lower temperature at the seal-cylinder interface; hence the temperature of cylinder C1 decreased.

### 3.3. Analysis of the Wear of Seals and Layers Formed on the Cylinders of Actuators

**Figure 8:**
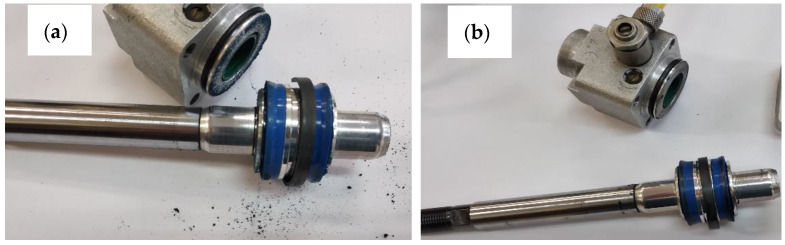
Image of the disassembled actuators (**a**) C1; (**b**) C2.

After the stand tests were completed, the test system was disassembled, and the macroscopically visible differences are shown in Figure 8a,b. After disassembling the cylinder with the C1 cylinder, wear products of the piston seals were noticed (Figure 8a). In the case of the C2 actuator, no such wear was noticed (Figure 8b).

**Figure 9:**
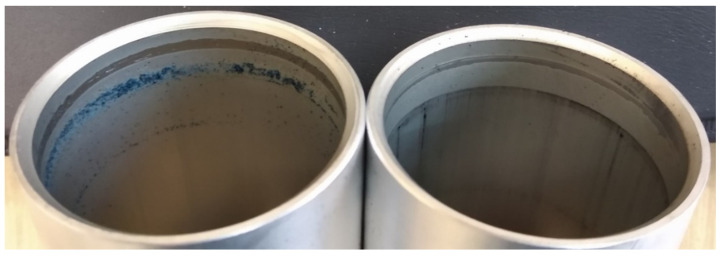
View of the cylinder C1 (**left side**) and C2 (**right side**).

Figure 9 shows images of the cylinders after disassembly of the system. The left side of the image shows the C1 cylinder with visible blue wear products from the polyurethane wiper seal. This effect is the result of significant resistance to movement and abrasive-adhesive wear of the seal in the cylinder C1, which in turn results from the lack of a lubricating layer that allows the wiper seals to slide on the cylinder surface.

**Figure 10:**
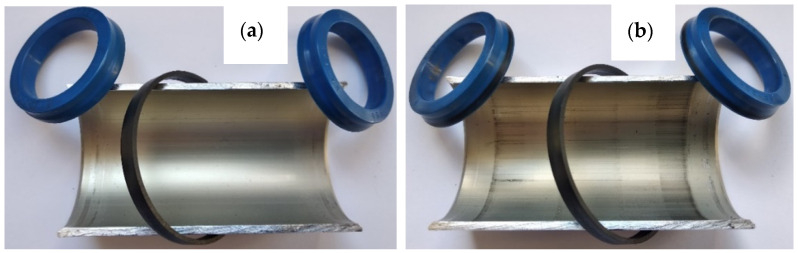
Cut cylinder C1 (**a**) and C2 (**b**) with the cooperating: piston seal (black) and scraper seals (blue).

Figure 10a,b show the images of cut cylinders and seals cooperating with their internal surfaces. The main macroscopic difference observed in this comparison is the presence of a black area both on the C2 cylinder surface and on the blue scraper seals cooperating therewith (Figure 10b).

**Figure 11:**
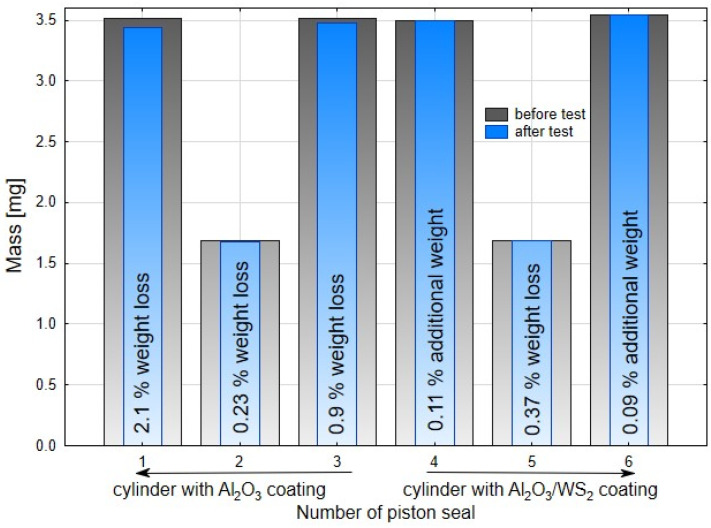
Mass diagrams of seals before and after the bench test.

The black area on the surface of cylinder C2, also visible in Figure 9 on the right, is the so-called sliding film. It was created as a result of cooperation of the sliding guide seal of the piston made of T5W material with the surface of the Al_2_O_3_/WS_2_ layer. The formation of such a sliding film in sliding associations operating under conditions of technically dry friction is closely related to the process of wear of the plastic and its transfer to the surfaces of the oxide layers. The cooperation of the scraper seals with the surface of the C2 cylinder resulted in the transfer of the T5W material also to the surfaces of the blue scraper seals, which resulted in the proper operation of the elements of the sliding system of this actuator. The application of the sliding film on the cylinder bearing surface C2 was possibly due to the prelubrication, which was most likely due to the addition of WS_2_ to the SFS electrolyte in the formation of the layer on the cylinder bearing surface. Solid lubricant in the form of WS_2_ introduced into the structure of the Al_2_O_3_/WS_2_ layer caused initial slip of the scraper seals on the oxide layer, reducing the movement resistance and wear of these seals, as was the case in the cylinder C1 of the actuator (Figure 9). Figure 11 shows the weight of the seals before (grey bars) and after (blue bars) the bench test. In the case of seals cooperating with the C1 cylinder, a weight loss was observed for all three seals. Approx. 2.1% weight loss was recorded for the left wiper seal, and approx. 0.9% of the initial mass for the right one. The products of this wear are shown in Figure 8a and Figure 9 (left cylinder). The piston guide seal lost approximately 0.23% of its original weight upon interaction with the C1 cylinder Al_2_O_3_ layer. In the case of the seals cooperating with the C2 cylinder, the weight was higher than the initial weight by about 0.11% for the left seal and 0.09% for the right wiper seal. It is related to the applied black layer of T5W material, shown in Figure 10b. The piston guide seal lost approximately 0.37% of its original weight upon engagement with the surface of the C2 cylinder.

The results of the weight tests carried out confirm the macroscopic observations after the completed stand tests.

### 3.4. Thickness, Structure and Surface Morphology of the Layers Produced on Actuator Cylinders

**Figure 12:**
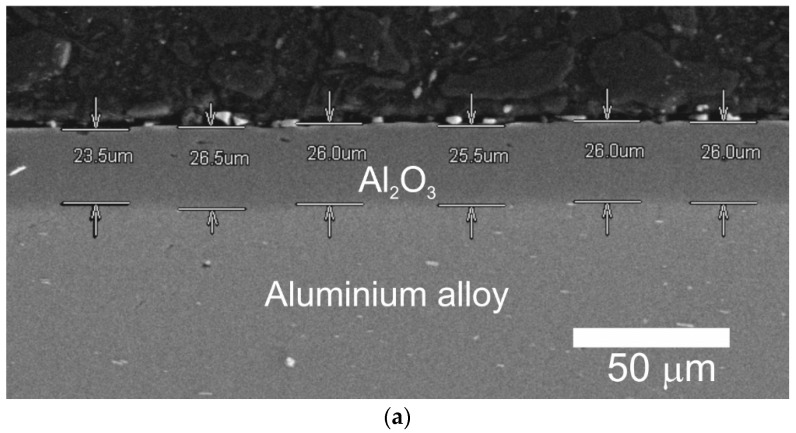

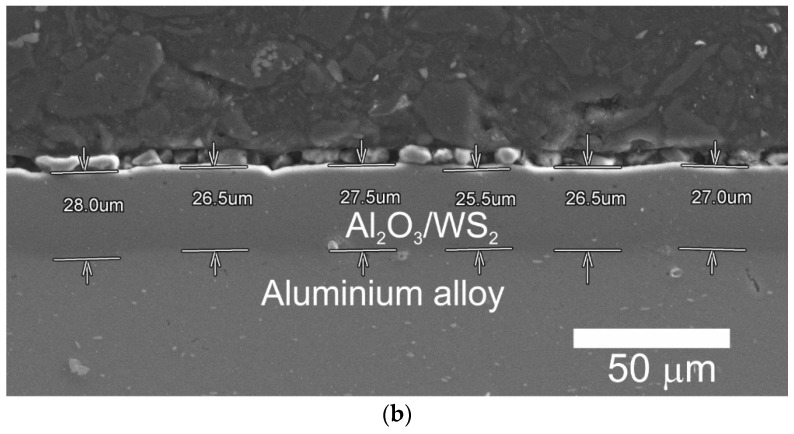
SEM images of C1 (**a**) and C2 (**b**) with thickness of coatings.

Figure 11 shows the thickness of the Al_2_O_3_ layer produced on the cylinder C1 (Figure 12a) and the thickness of the Al_2_O_3_/WS_2_ layer produced on the cylinder C2 (Figure 12b). The layer thickness on cylinder C1 was approximately 25.58 ± 1.07 μm, and on cylinder C2 approximately 26.83 ± 0.87 μm.

Taking into account the error resulting from the measurement method and the standard deviation, it can be concluded that both layers were of similar thickness. The thickness of the Al_2_O_3_ layer formed on the substrates of aluminum alloys, equal to about 25 μm, is sufficient for tribological cooperation in systems of technically dry friction with plastics.

**Figure 13:**
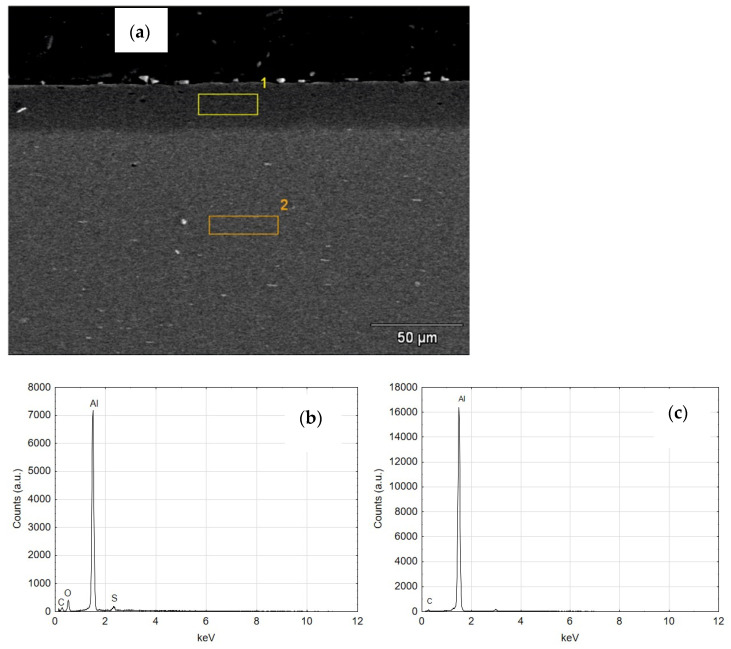
(**a**) SEM image of the structure of C1 cylinder (**b**) EDS spectrum of area 1 (**c**) EDS spectrum of area 2.

Figure 13a shows a SEM photo of a transverse view of cylinder 1 with the marked areas indicating the location of the SEM/EDS analysis. As can be seen from the spectrum of the first area in Figure 13b, the darker area on the cross-section is the aluminum oxide layer, while the lighter area is the base of the layer, i.e., aluminum alloy Figure 13c.

**Figure 14:**
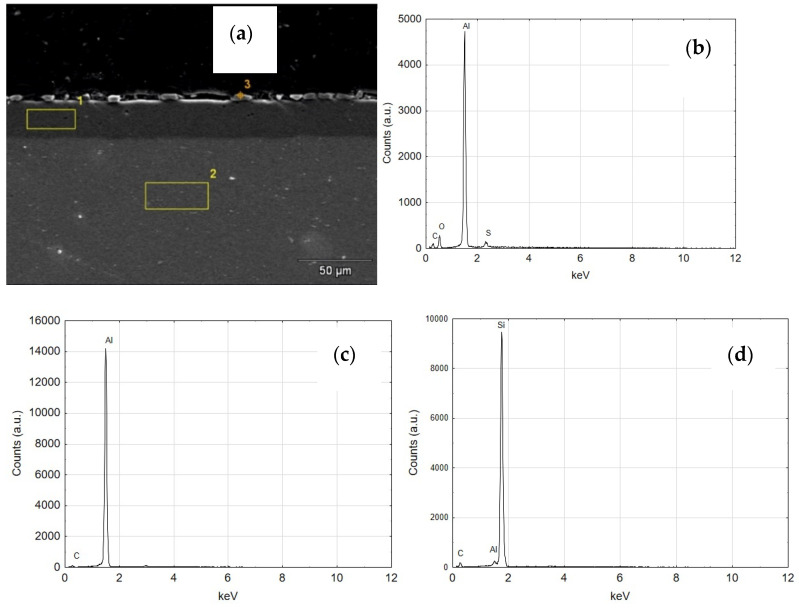
(**a**) SEM image of the structure of C2 cylinder (**b**) EDS spectrum of area 1 (**c**) EDS spectrum of area 2, (**d**) EDS spectrum of area 3.

Figure 14a shows a SEM photo of the C2 cylinder cross-section with the three SEM/EDS analysis areas marked. The first area was characterized during the qualitative analysis as an Al_2_O_3_ oxide layer—Figure 14b, the second area as an aluminum alloy—Figure 14c. Area 3 indicates silicon particles—Figure 14d from grinding the cross-sections that got between the tested cylinder and the resin in which the sample was incorporated.

**Figure 15:**
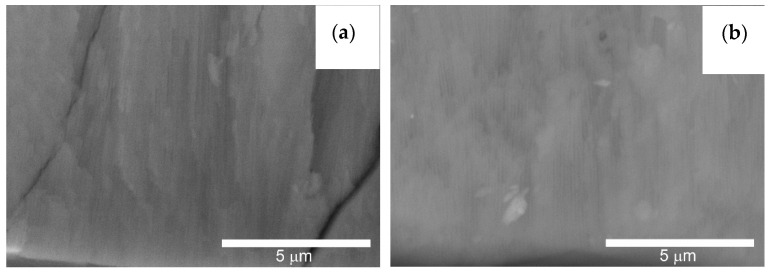
SEM images of the fresh structure: (**a**) C1 cylinder, (**b**) C2 cylinder—magnification 10,000×.

Figure 15 shows the fractures of the layers formed on the cylinder faces. On both the cylinder C1 and C2, the typical structure of longitudinal Al_2_O_3_ fibers can be observed.

Al_2_O_3_ fibers are formed during the anodizing process, creating free spaces between them, necessary for the movement of oxygen ions connecting with the anode material.

**Figure 16:**
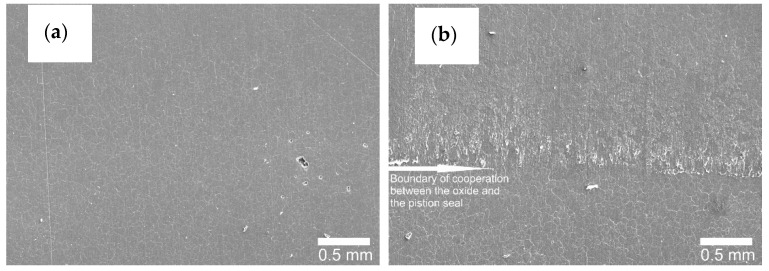
SEM images of the surface after tribological test: (**a**) C1 cylinder, (**b**) C2 cylinder—magnification 35×.

**Figure 17:**
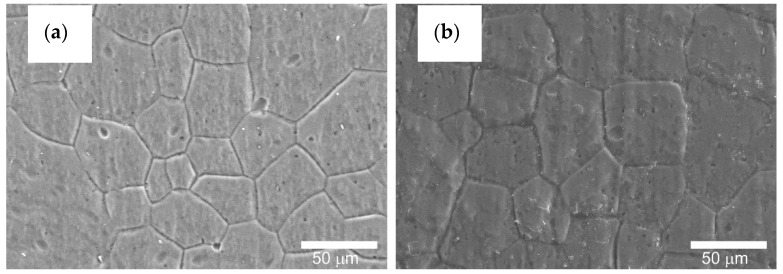
SEM images of the surface after tribological test: (**a**) C1 cylinder, (**b**) C2 cylinder—magnification 500×.

**Figure 18:**
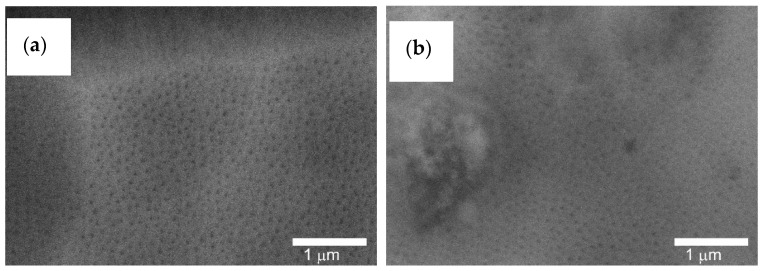
SEM images of the surface after tribological test: (**a**) C1 cylinder, (**b**) C2 cylinder—magnification 25,000×.

Figure 16, Figure 17 and Figure 18 show the cylinder bearing surface after the operational test. Figure 16b shows a clear border between the area of tribological cooperation of the wiper seal with cylinder C2, and the area where the layer from outside this area can be observed. Part of the cooperation area in the case of the C2 cylinder is characterized by an applied sliding film, clearly visible as a black area in Figure 10b. This phenomenon is not observed in the case of the C1 cylinder, which also confirms the macroscopic observations. The images also show a mesh of grains reflecting the microstructure of the aluminum alloy substrate, which is also shown in Figure 17 at a magnification of 500×. Figure 18a,b in the magnification of 25,000×, the nanoporous surface morphology typical for the Al_2_O_3_ oxide layers was observed. As our research has shown for the Al_2_O_3_ layer thickness of approx. 25 μm, apart from the typical surface morphology visible at high magnifications, at lower magnifications it is possible to perfectly observe the microstructure of the aluminum alloy substrate, on which the amorphous aluminum oxide layer grows. The boundaries of aluminum grains become the source of unevenness formed on the surface of the aluminum oxide.

The visible grain boundaries on the layers are the result of the action of KOH and HNO_3_ solutions on the alloy surface and the inheritance of the substrate characteristics by the forming Al_2_O_3_ layers. Such a phenomenon has also been confirmed in research [35].

### 3.5. Spectrometric and EDS Analysis of the Layers Produced on the Cylinders of the Actuators

**Figure 19:**
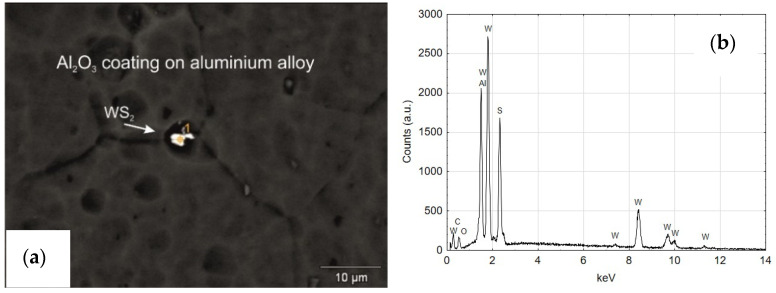
(**a**) SEM YAGBSE image of C2 cylinder, (**b**) EDS spectrum of area 1.

**Figure 20:**
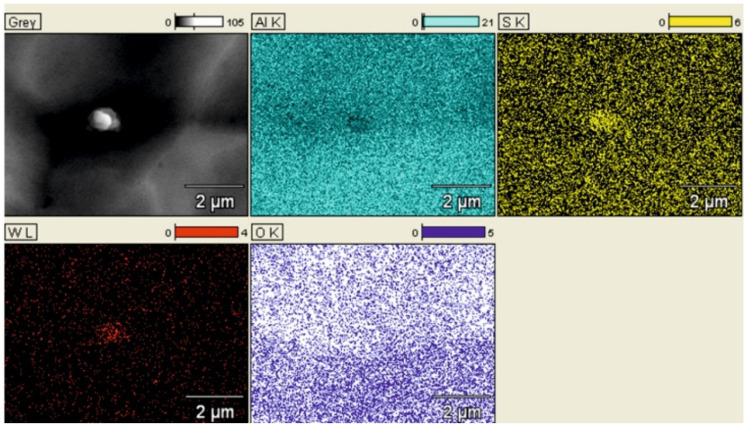
SEM/EDS mapping of the coating of C2 cylinder with visible WS_2_ powder built in the microstructure of Al_2_O_3_ oxide.

Figure 19a shows a SEM YAGBSE image made with the use of backscattered electrons. Only with this type of identification it was possible to reveal the modifier present on the surface of the oxide layer in the form of tungsten disulfide WS_2_ powder—Figure 19b. Figure 20 shows the SEM/EDS mapping of the C2 cylinder surface with visible WS_2_ powder embedded in the microstructure of the Al_2_O_3_ oxide layer. The figure shows the share of individual elements Al, S, W and O in the scanned area.

**Figure 21:**
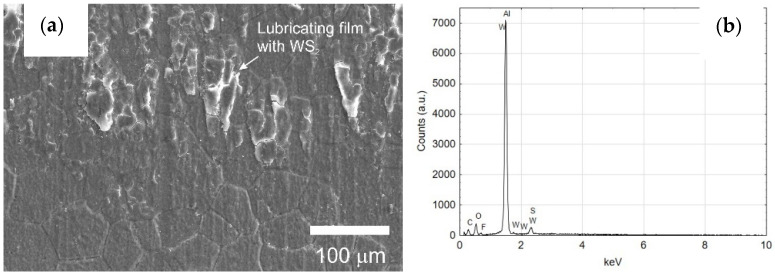
(**a**) The surface of the Al_2_O_3_ coating (C2 cylinder) at the border of tribological cooperation with the applied sliding film containing WS_2_ powder, (**b**) EDS spectrum of the area indicated in (**a**) image.

**Figure 22:**
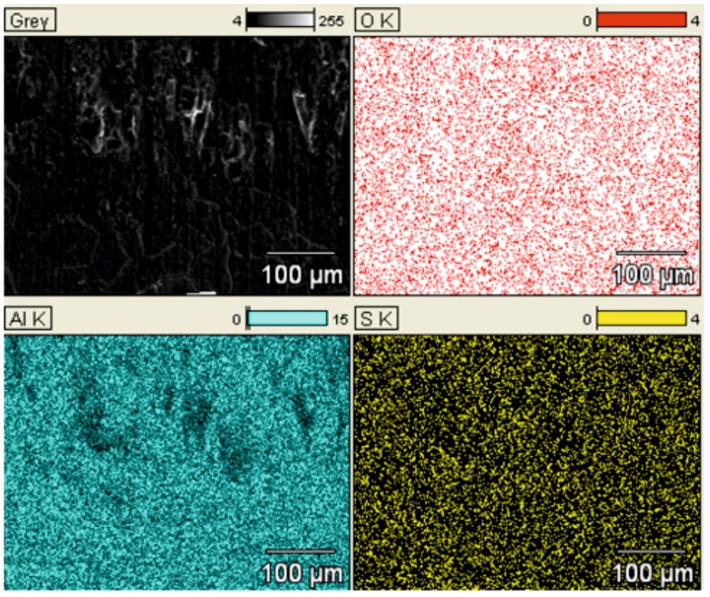
SEM/EDS mapping of the coating of C2 cylinder at the border of tribological cooperation.

The qualitative EDS analysis was also carried out for the skid film formed on the inner surface of the cylinder C2—Figure 21. The SEM/EDS image mapping (Figure 22) shows no tungsten in the elemental fraction in the scanned area. Limiting the scanned area for EDS analysis to a small area indicated in Figure 21a allows to reveal the presence of tungsten disulfide in the formed sliding film (Figure 21b). As the research carried out shows, in order to reveal the presence of WS_2_ lubricant in the sliding film deposited on the Al_2_O_3_ layer, it is necessary to conduct an in-depth qualitative analysis of SEM/EDS. Mapping itself is not sufficient in this case.

**Figure 23:**
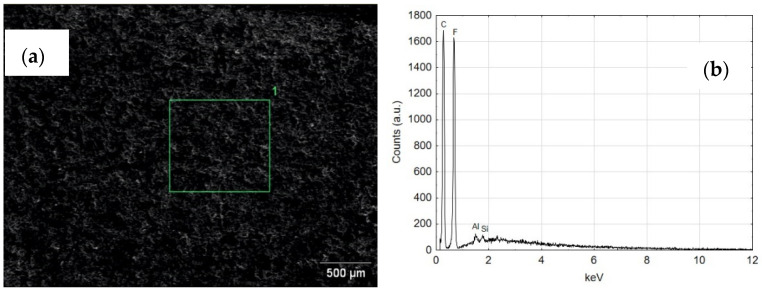
(**a**) SEM image of the guiding seal cooperating with C1, (**b**) EDS spectrum of area 1.

Figure 23 shows the SEM and EDS of the guiding seal surface associated with the cylinder face C1. The high carbon and fluorine reflex is characteristic of the T5W material, from which the black guide seal is made. The EDS tests carried out for the seal cooperating with the C2 cylinder surface show similar results of the elemental composition.

**Figure 24:**
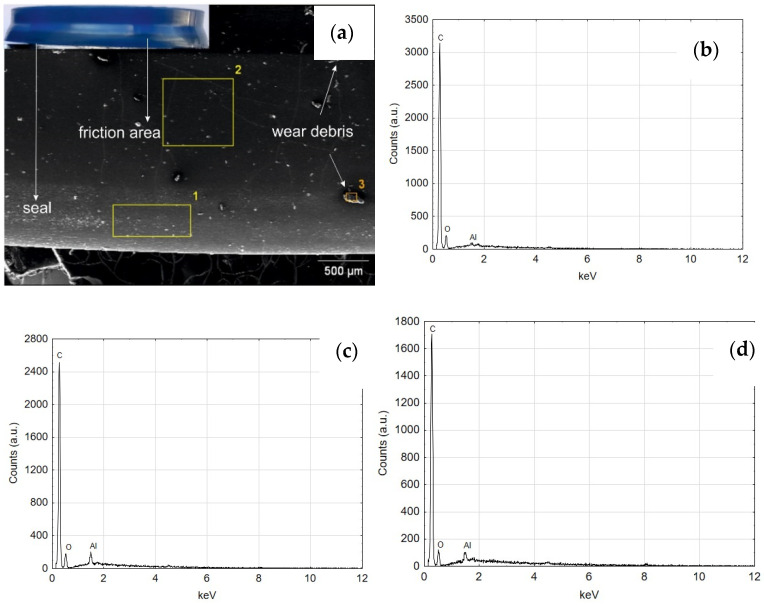
(**a**) SEM image of the seal cooperating with C1, (**b**) EDS spectrum of area 1 (**c**) EDS spectrum of area 2, (**d**) EDS spectrum of the area 3.

**Figure 25:**
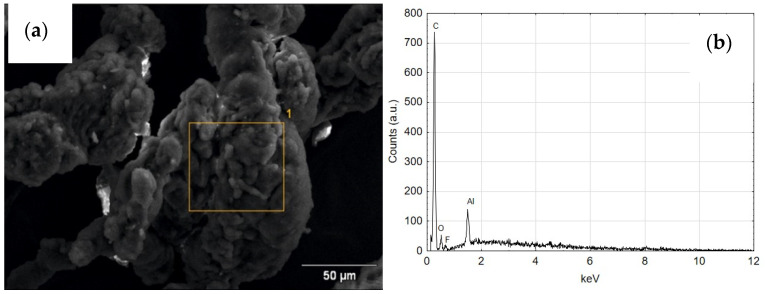
(**a**) SEM image of the wear debris due to the friction of the seal and C1 cylinder, (**b**) EDS spectrum of area 1.

Figure 24 shows the SEM and EDS photos of the wiper seal cooperating with the surface of the cylinder C1. Both regions 1 and 2 reveal the elemental composition corresponding to the material of the wiper seal (Figure 24b,c). The reflections from aluminum indicate that wear products appear on the seal, originating from the Al_2_O_3_ oxide layer and the wiper seal (Figure 24d). The image of the wear product resulting from the cooperation of the seal with the C1 cylinder surface is shown in Figure 25a, its qualitative analysis EDS in Figure 25b. The artefact visible in the image is a conglomerate of wear products of the wiper seal and the Al_2_O_3_ aluminum oxide layer.

**Figure 26:**
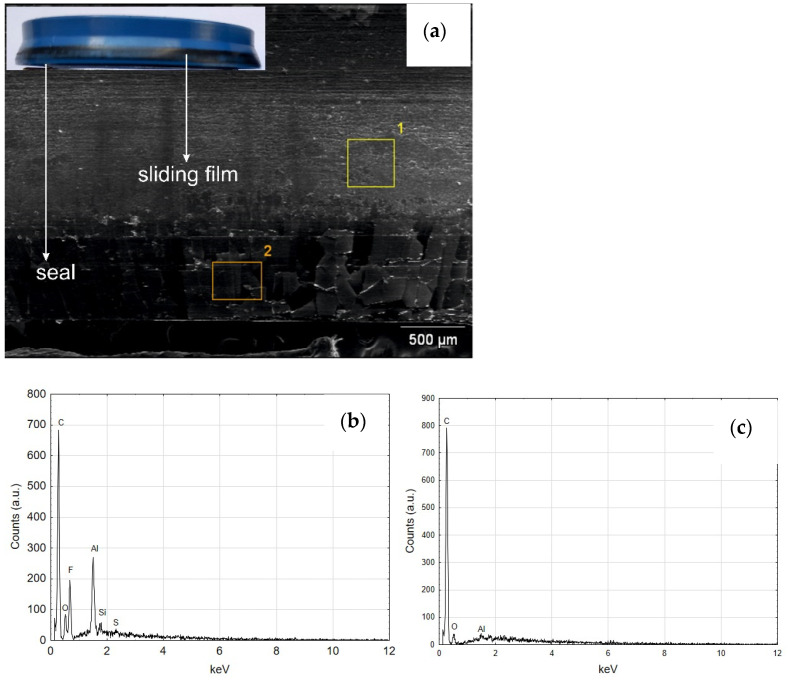
(**a**) SEM image of the seal cooperating with C2 cylinder with visible area of the sliding film, (**b**) EDS spectrum of area 1 (**c**) EDS spectrum of area 2.

**Figure 27:**
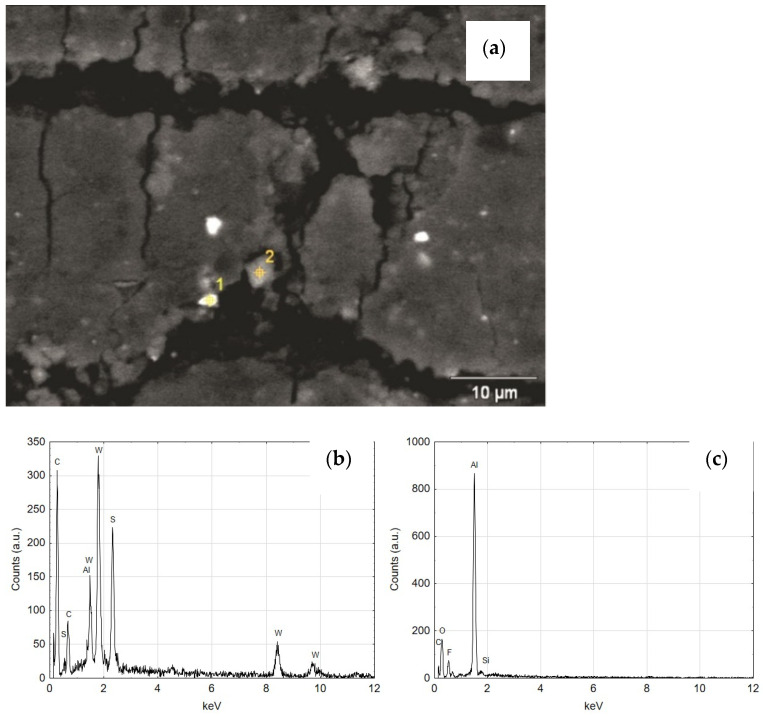
(**a**) SEM image of magnified area of the sliding film created on the seal cooperating with C2 cylinder, (**b**) EDS spectrum of area 1, (**c**) EDS spectrum of area 2.

Figure 26a shows the SEM of the areas of the wiper seal cooperating in the running surface of the cylinder C2 with the sliding film applied. As shown by the EDS analysis, the sliding film applied to the seal consists of the elements of the wiper seal material, the piston guide seal and the Al_2_O_3_ layer wear products (Figure 26b and Figure 27c). The tests carried out for the area shown in also reveal that there are WS_2_ lubricant particles embedded in the sliding film on the wiper seal (Figure 27b). As shown in Figure 10a,b as well as Figure 24a and Figure 26a, the traces resulting from tribological cooperation are different. It is assumed that the surface of the C2 cylinder has a more sliding character of friction, and the slip between the surfaces of the cooperating elements is improved by the presence of WS_2_ lubricant.

### 3.6. Stereometric Analysis of the Surface of the Layers Produced on the Actuator Cylinders

**Figure 28:**
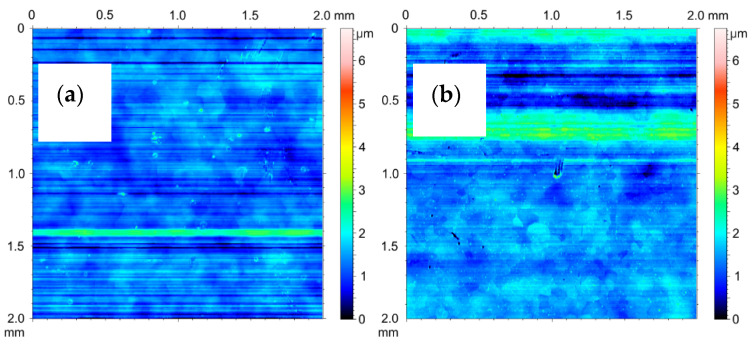
SGP 2D images of the cylinder (**a**) before etching, (**b**) after etching.

**Figure 29:**
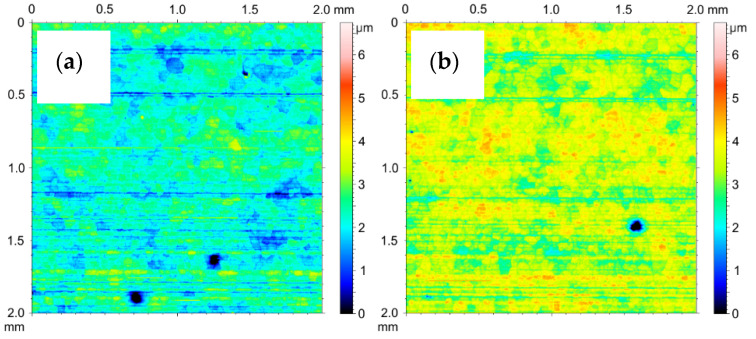
SGP 2D images of the cylinder C1: (**a**) nonfriction area, (**b**) tribological cooperation area.

**Figure 30:**
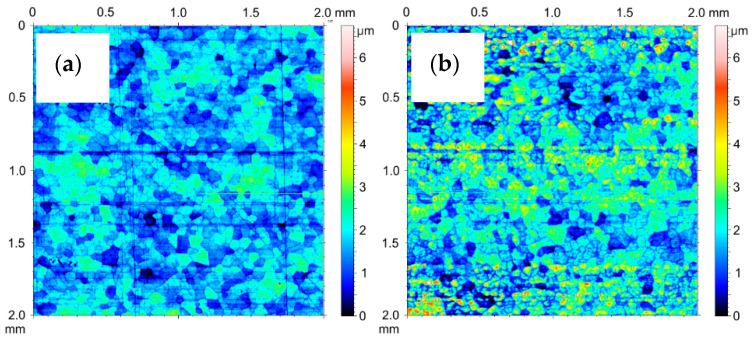
SGP 2D images of the cylinder C2: (**a**) nonfriction area, (**b**) tribological cooperation area.

The 2D stereometric image of the surface of the material from which the test cylinders were cut out is shown in Figure 28a. The transverse scratches visible on it are characteristic of the technological process of manufacturing the pipe from which the cylinders were prepared. Under the strip of the horizontal irregularities, a delicate mesh of aluminum alloy grains can be seen. Figure 28b shows the inner surface of the cylinder after etching, prepared for the electrochemical oxidation process. As a result of etching in a 2D image, the mesh of aluminum alloy grains is more noticeable. Figure 29a shows 2D image of oxide layer formed on the inner faces of cylinder C1 after electrochemical treatment. Figure 29b shows 2D images of oxide layers on cylinder C1 after tribological cooperation. Figure 30a shows 2D image of oxide layer formed on the inner faces of cylinder C2 after electrochemical treatment. Figure 30b shows2D image of oxide layer on cylinder C2 after tribological cooperation. The obtained 2D images, in addition to the range of unevenness of the tested surfaces, show the microstructure of the substrate, which is a mesh of aluminum alloy grains.

**Figure 31:**
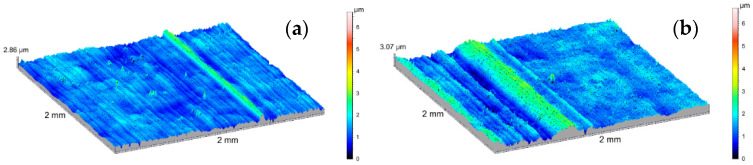
SGP 3D images of the cylinder (**a**) before etching, (**b**) after etching.

**Figure 32:**
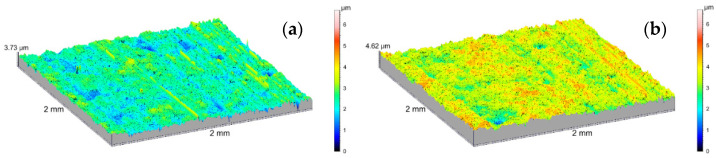
SGP 3D images of the cylinder C1: (**a**) non-friction area, (**b**) tribological cooperation area.

**Figure 33:**
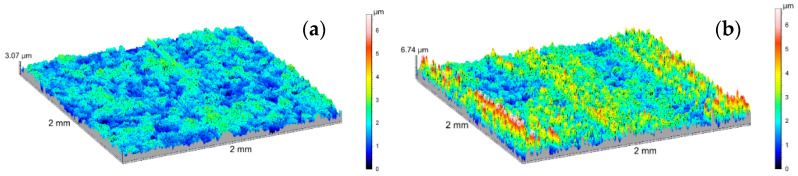
SGP 3D images of the cylinder C2: (**a**) non-friction area, (**b**) tribological cooperation area.

**Figure 34:**
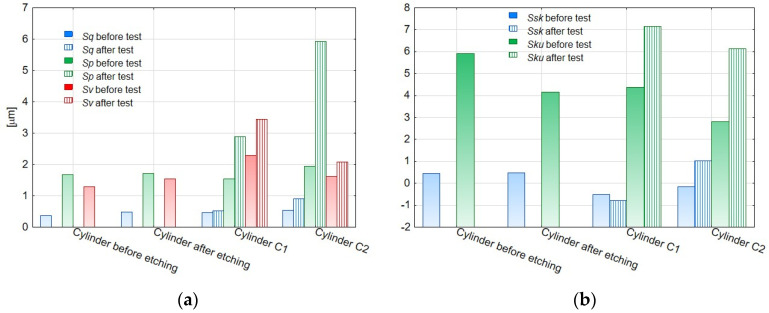
(**a**,**b**) The amplitude parameters of the surface geometrical structure of the samples before and after the test.

The amplitude parameters of the tested surfaces are summarized in Table 1. The 3D visualization of the geometric surface of the measured elements is shown in Figure 31, Figure 32 and Figure 33. The amplitude parameters of the tested surfaces are also partially shown in the graphs—Figure 34. The analysis of the surface topography showed that the starting material was characterized by the lowest parameter of the mean surface roughness deviation *Sq* = 0.36 μm, and etching preparing the surfaces for oxidation increased this parameter to the value of *Sq* = 0.47 μm. Oxidation of the C1 cylinder surface in the SFS electrolyte did not significantly affect the average the *Sq* value, which was 0.46 μm, while the oxidation of the C2 cylinder in the SFS electrolyte with WS2 addition slightly increased the *Sq* value to 0.54 μm. The parameters *Ssk* and *Sku* determine the asymmetry (skew) and surface slope (kurtosis), respectively, and are sensitive to large individual extremes. Negative values of the skewness coefficient *Ssk* = −0.5 and *Ssk* = −0.15 were obtained for the smooth surfaces of C1 and C2 cylinders, respectively, before tribological cooperation. After tribological cooperation, this parameter changed and was, respectively, for C1: *Ssk* = −0.78 and for C2: *Ssk* = 1.02. The negative skewness of the examined layers indicates the surface of the layers of a plateau character, which is well visible in the isometric Figure 32a,b and Figure 33a. Kurtosis values were respectively *Sku* = 4.38 µm and *Sku* = 2.8 µm for C1 and C2 before the test and *Sku* = 7.16 µm and *Sku* = 6.12 µm for C1 and C2 after the tribological test. The increase in the Sku parameter measured for the surface after the tribological test proves that the tribological cooperation of both the C1 and C2 cylinder smooth surfaces resulted in an increase in the occurrence of high and sharp peaks, which is particularly visible in Figure 33b.

The analysis of the parameters *Sp* and *Sv* or *Sp* and *Sz* gives information about the shape of the profile and allows to conclude on the abrasion resistance of the tested surface. Prior to the test, the C1 cylinder smoothing was characterized by a void coefficient *Sp*/*Sz* << 0.5 (Table 1), which suggests that the surface irregularities of the Al_2_O_3_ layer were characterized by rounding of the peaks, which in turn increased its abrasion resistance. In the case of the C2 cylinder bearing surface, the ratio *Sp*/*Sz* > 0.5 (Table 1), indicating that its initial abrasion resistance was lower. After the tribological test, both the C1 and C2 cylinders were characterized by a void ratio of *Sp*/*Sz* >> 0.5; therefore the abrasion resistance of the surfaces of both cylinders decreased.

The surface of cooperation after the stand test for the C2 cylinder was characterized by a higher roughness (*Sq*) and skewness (*Ssk*) parameter, a slightly higher void filling parameter (*Sv*/*Sz*) and a higher parameter of the average absolute values of the heights of the five highest peaks and five lowest pits within the sampling area (*Sz*), compared with the bore surface of the C1 cylinder. The properties of the surface of the C2 cylinder bearing surface observed in the isometric image (Figure 33b) after the tribological test are related to the created sliding film, which influenced the unidirectional nature and high density of the peaks of this surface.

**Figure 35:**
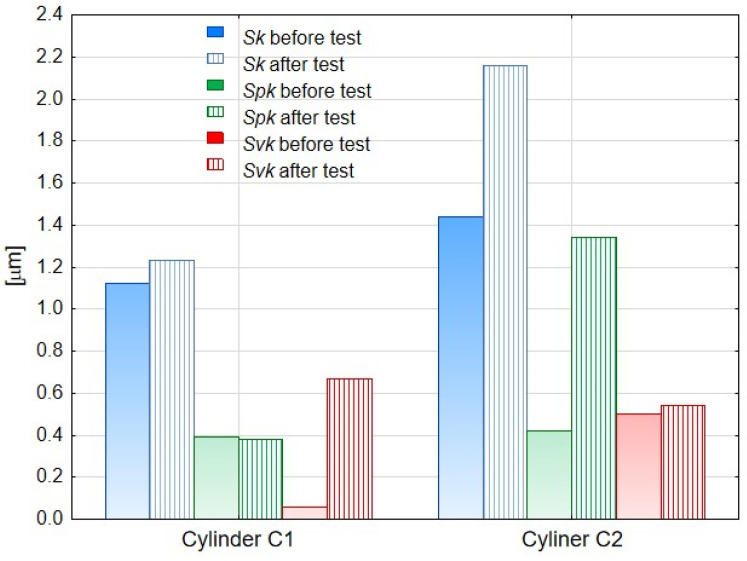
Parameters of the Abbott–Firestone curve before and after tribological test for the analyzed cylinders *Sk* (core roughness depth), *Spk* (reduced peak height), and *Svk* (reduced valley depth).

Table 2 presents the parameters of the Abbott–Firestone curve determined for the surface of the cylinder lining surfaces before and after the bench test, and in Figure 35 their values are visualized. For both the C1 and C2 cylinder, an increase in the value of the *Sk* parameter was recorded after the test. The *Sk* parameter describes the nominal roughness (roughness height of the core) and can be a measure of the effective roughness depth after the initial run-in period. The *Spk* parameter determines the resistance of the surface to abrasion, and its low values prove its high resistance. According to the obtained result, the C1 cylinder showed higher abrasion resistance after the test. This is most likely due to the fact that a sliding film had formed on the surface of the cylinder C2, which made the piston seals slide better during operation of the cylinders. Simultaneously the abrasion resistance thereof was lower. The S*vk* parameter, in turn, is a measure of the lubricant retention capacity of the sliding surfaces. This parameter was higher after friction for the C1 cylinder bearing surface. We explain this behavior as follows: the low value of the S*vk* parameter for the C1 cylinder after oxidation is related to the plato-type surface, on which, as a result of cooperation with the wiper seal, deeper grooves were formed, which, however, were not filled with a sliding film, as in the case of the modified layer WS_2_ on the cylinder C2 surface.

## 4. Conclusions

The research results and their analysis presented in the article confirm the sense of modifying the Al_2_O_3_ layers with tungsten disulfide 2H-WS_2_ in order to increase the service life of pneumatic cylinders with oxide finish, supplied with air not lubricated with oil mist. Modification of the Al_2_O_3_ layer with tungsten disulfide contributed to the formation of a sliding film on the C2 cylinder surface and wiper seals cooperating with it, which translated into uniform piston operation during 180 h of cylinder operation. The cylinder with the unmodified layer showed irregular operation after about 70 h. Increased resistance to piston movement caused by the lack of lubrication in the C1 cylinder, contributed to excessive wear of the wiper seals and the piston guide ring as a result of tribological cooperation. The greater resistance to motion was also the reason for the higher temperature in the friction zone of this actuator. The better sliding properties of the C2 cylinder smoothing surface result from the presence of the 2H-WS_2_ tungsten disulfide dispersion on the surface of the layer produced by anodizing aluminum alloy in an electrolyte with the addition of this solid lubricant. The presence of WS_2_ on the surface of the oxide layer facilitates the overlapping of the sliding film on the cooperating elements and allows the nature of the operation of the sliding pair to be changed. Despite the higher values of the surface roughness parameters shown for the C2 cylinder bearing surface, the geometric structure of the surface of the WS_2_ modified layer remains favorable for tribological applications in oil-free kinematic systems.

## Figures and Tables

**Table 1:** The amplitude parameters of the surface geometrical structure of cylinder before and after the test.

Sample	SGP Parameters							SGP Parameters						
	*Sq* μm	*Ssk*	*Sku*	*Sp* μm	*Sv* μm	*Sz* μm	*Sp*/*Sz*	*Sq* μm	*Ssk*	*Sku*	*Sp* μm	*Sv* μm	*Sz* μm	*Sp*/*Sz*
	Before test							After test						
**Before etching**	0.36	0.44	5.93	1.68	1.30	2.91	0.58	-	-	-	-	-	-	-
**After etching**	0.47	0.47	4.16	1.71	1.54	3.18	0.54	-	-	-	-	-	-	-
**C1**	0.46	−0.5	4.38	1.54	2.29	3.70	0.42	0.51	−0.78	7.16	2.88	3.44	3.97	0.72
**C2**	0.54	−1.15	2.8	1.95	1.62	3.49	0.56	0.9	1.02	6.12	5.92	2.08	7.76	0.76

**Table 2:** The Abbott–Firestone curve parameters before and after tribological interaction for measured specimens.

Sample	*Sk* [μm]		*Spk* [μm]		*Svk* [μm]	
	before test	after test	before test	after test	before test	after test
C1	1.12	1.23	0.39	0.38	0.58	0.67
C2	1.44	2.16	0.42	1.34	0.50	0.54

## Data Availability

The data presented in this study are available on request from the corresponding author.
